# Insulin Resistance and Metabolic Dysfunction in Early-Stage Parkinson’s Disease: Evidence from a Preliminary Case-Control Study

**DOI:** 10.3390/jcm15031021

**Published:** 2026-01-27

**Authors:** Elena Contaldi, Lorenzo Ciocca, Francesco Mignone, Michela Barichella, Alessia Siribelli, Giulia Lazzeri, Ioannis Ugo Isaias, Gianni Pezzoli, Federica Invernizzi

**Affiliations:** 1 Parkinson Institute of Milan, ASST Gaetano Pini-CTO, 20126 Milan, Italy; elena.contaldi@asst-pini-cto.it (E.C.); giulia.lazzeri@asst-pini-cto.it (G.L.); ioannis.isaias@asst-pini-cto.it (I.U.I.); gianni.pezzoli@gmail.com (G.P.); 2 Center for Liver Disease, Division of Internal Medicine and Hepatology, IRCCS Ospedale San Raffaele, 20132 Milan, Italy; siribelli.alessia@hsr.it (A.S.); invernizzi.federica@hsr.it (F.I.); 3 Internal Medicine Department, Vita-Salute San Raffaele University, 20132 Milan, Italy; f.mignone@studenti.unisr.it; 4 Clinical Nutrition Unit, ASST Gaetano Pini-CTO, 20126 Milan, Italy; barichella@parkinson.it; 5 Neurology Department, University Hospital of Würzburg, Julius Maximilian University of Würzburg, 97070 Würzburg, Germany; 6 Fondazione Pezzoli per la Malattia di Parkinson, 20125 Milan, Italy

**Keywords:** Parkinson’s disease, insulin resistance, metabolic dysfunction

## Abstract

**Background:** Parkinson’s disease (PD) is increasingly recognized as a multisystem disorder in which metabolic dysfunction may contribute to disease susceptibility and progression. Peripheral insulin resistance (IR) has been implicated in PD, but data in levodopa-naïve patients are currently limited. Objective: To investigate the prevalence of IR and metabolic dysfunction in early-stage, levodopa-naïve PD patients and their association with clinical features. **Methods:** We conducted an exploratory case–control study including 20 levodopa-naïve PD patients and 40 age-, sex-, and BMI-matched healthy controls. Participants underwent comprehensive clinical and metabolic assessments, including fasting glucose, insulin, lipid profiles, and HOMA-IR calculation. Peripheral IR was defined using HOMA-IR cut-offs of ≥2.0 (primary analysis) and ≥2.5 (sensitivity analysis). ANCOVA adjusted for age, sex, and BMI was used for between-group comparisons. **Results:** PD patients exhibited higher fasting insulin (10.7 ± 5.2 vs. 8.0 ± 4.4 µIU/mL; *p* = 0.020) and HOMA-IR (2.63 ± 1.40 vs. 1.89 ± 1.21; *p* = 0.014) compared to controls. Using a HOMA-IR ≥ 2.0, IR prevalence was 70% in PD vs. 32.5% in controls (OR = 4.85, 95% CI 1.52–15.50, *p* = 0.012). ANCOVA analysis confirmed group differences after adjusting for covariates (respectively, *p* = 0.032 for insulin and *p* = 0.023 for HOMA-IR). A sensitivity analysis excluding six patients receiving dopaminergic therapy further supported the robustness of the results. No significant correlations were observed between IR and disease severity scores. **Conclusions:** Early-stage, levodopa-naïve PD patients exhibit a higher prevalence of peripheral insulin resistance compared with matched controls. These findings support the hypothesis that metabolic dysfunction is an intrinsic component of PD pathophysiology and may represent a target for early intervention.

## 1. Introduction

Parkinson’s disease (PD) is among the most prevalent neurodegenerative conditions worldwide, ranking after Alzheimer’s disease (AD) in global prevalence, classically characterized by degeneration of dopaminergic neurons in the substantia nigra and pathological aggregation of α-synuclein [1]. While these hallmarks explain the core motor syndrome, PD is recognized as a multisystem condition with prominent non-motor features, including cognitive impairment, sleep and autonomic disturbances, gastrointestinal dysfunction, and metabolic alterations. This broader clinical perspective mirrors a complex pathophysiology in which neuronal injury intersects with systemic processes extending beyond the basal ganglia [1]. Converging data indicate that metabolic dysfunction (notably insulin resistance, dyslipidemia, mitochondrial dysfunction, and chronic low-grade inflammation) may influence both susceptibility to PD and disease progression. Epidemiological studies have consistently associated type 2 diabetes mellitus (T2DM), obesity, and metabolic syndrome with a higher risk of incident PD and worse clinical trajectories among those already affected [2,3,4].

Impaired insulin signalling in the central nervous system affects neuronal glucose metabolism, synaptic plasticity, and dopamine homeostasis, potentially promoting α-synuclein misfolding and aggregation [5]. In parallel, deficits in mitochondrial oxidative phosphorylation with excessive reactive oxygen species (ROS) generation can increase dopaminergic vulnerability through the amplification of oxidative stress and neuroinflammation [6,7]. These insights support the hypothesis that PD, besides being a synucleinopathy, may also be viewed as a disorder of impaired energy metabolism. The “type 3 diabetes” framework [8], initially advanced for AD, has increasing relevance to PD, where neuronal insulin resistance and defective pathways of mitochondrial quality control (e.g., PINK1/Parkin-mediated mitophagy) may converge to accelerate neurodegeneration [5]. Systemic metabolic alterations can further amplify central injury through peripheral-to-brain inflammatory signalling, while PD-related autonomic impairment, reduced physical activity, and altered energy balance may worsen metabolic homeostasis—establishing a vicious bidirectional cycle [9]. Beyond pathobiology, this neuro-metabolic interface has translational implications. Several metabolic therapies—including GLP-1 receptor agonists and metformin—have shown preliminary neuroprotective signals in preclinical and early clinical studies, potentially via improvements in insulin signalling, mitochondrial efficiency, and neuroinflammation [10,11]. Likewise, lifestyle interventions such as structured exercise [12] and Mediterranean-style nutrition [13] improve metabolic health and have documented symptomatic benefits in PD, with mounting interest in their disease-modifying potential. Growing evidence implicates gut–brain communication and microbiome shifts in modulating both immune–inflammatory pathways and energy metabolism, offering additional modifiable targets [14]. Despite this growing literature, key knowledge gaps remain. First, the relative contributions of distinct metabolic domains (e.g., glycemic control, lipid profiles, adipokines) to PD risk and progression are incompletely disentangled and may vary across clinical phenotypes [15]. Second, many existing studies rely on observational designs, inconsistent definitions of metabolic dysfunction, and are vulnerable to confounding factors, as well as potential metabolic effects of dopaminergic treatment [16]. Third, there is a need for integrated analyses that relate quantitative metabolic markers to clinically meaningful outcomes (motor severity, cognition, progression rates), ideally with adjustment for demographic, vascular, and treatment confounders [17]. To address these gaps, we conducted an exploratory, case–control study of early-stage, levodopa-naïve PD patients and matched non-PD controls. We examined predefined metabolic measures, focusing on insulin resistance and lipid profiles. Our primary aim was to assess whether PD is associated primarily with an increased burden of insulin resistance and secondarily with other markers of metabolic disorders. Furthermore, we explored whether greater insulin resistance is associated with increased disease severity.

## 2. Materials and Methods

**Study design and participants**: We conducted a case–control observational study between September 2024 and September 2025 at the Parkinson Institute of Milan, ASST Gaetano Pini-CTO (a tertiary referral centre for PD). We consecutively enrolled 20 patients with PD who were levodopa-naïve. PD diagnosis was established by movement disorder specialists according to the Movement Disorder Society clinical diagnostic criteria [18]. Inclusion criteria were age > 50 years, ability to provide informed consent, and no previous exposure to levodopa. The age threshold was chosen to minimize the likelihood of including individuals with monogenic or early-onset PD, who may display distinct genetic and metabolic profiles compared to typical late-onset cases [19]. Levodopa-treated patients were excluded to avoid potential confounding effects of dopaminergic therapy on carbohydrate metabolism; exposure to non-ergot-derived dopamine agonists (DAs) or MAO-B inhibitors (MAO-BIs) was permitted. Indeed, improved insulin-sensitivity has been reported in ergot-derived dopamine agonists [20], whereas evidence for non-ergot-derived DAs and MAO-BIs is inconclusive and mostly derived from preclinical models [21,22].

Main exclusion criteria were atypical/secondary parkinsonism, other major neurological disorders, acute systemic illness, diagnosis of diabetes, a history of endocrine and metabolic disorders linked to IR (including primary hyperaldosteronism, acromegaly, polycystic ovary syndrome and obstructive sleep apnea), and current use of medications known to markedly affect glucose/lipid metabolism (e.g., insulin, metformin, GLP-1 receptor agonists, SGLT2 inhibitors, systemic corticosteroids). For comparison, we recruited 40 healthy individuals attending routine health assessments in the preventive medicine programme at IRCCS San Raffaele. Healthy controls (HC) were matched 2:1 to PD cases by sex, age (±3 years), and by BMI (±2 kg/m^2^). Eligibility criteria for controls included no history of overt diabetes or other major endocrine disorders associated with IR; we also excluded subjects with a history of dopaminergic therapy and those taking medications with major metabolic effects. All participants underwent a structured baseline assessment including demographic and anthropometric data, comorbidities, current medications, dietary habits, and physical activity. Study procedures were approved by the local Ethics Committee (Comitato Etico Territoriale Lombardia 3, protocol 3499/23), and this research was conducted in accordance with the principles of the Declaration of Helsinki and its subsequent amendments.

**Clinical and metabolic assessment:** All participants underwent a comprehensive neurological evaluation with a focus on metabolic status. Neurological assessment included the Unified Parkinson’s Disease Rating Scale UPDRS (Parts I–III) [23] and Hoehn & Yahr staging [24] performed by movement disorder specialists. Anthropometric data (height, weight, BMI) were collected by trained staff. Laboratory evaluation comprised fasting glucose, HbA1c, fasting insulin, lipid profile (total cholesterol, HDL-C, LDL-C, triglycerides), liver/kidney function tests, and thyroid/vitamin panels. C-reactive protein (CRP) was collected as part of a broader inflammatory biomarker panel; however, CRP measurements were not available for all participants and were therefore not included in the analysis. Blood samples were collected at the same centre and analyzed using standardized assays, ensuring comparability across participants. To limit potential acute pharmacodynamic effects on metabolic measures, all PD patients on treatment with non-ergot-derived DAs and MAO-BIs underwent overnight medication suspension (≥12 h) before fasting blood withdrawal.

The Homeostasis Model Assessment for Insulin Resistance (HOMA-IR) was calculated as fasting insulin (µU/mL) × fasting glucose (mg/dL)/405; a value ≥ 2.0 was the prespecified threshold for insulin resistance based on previous evidence in PD [25] and data from ClinicalTrials.gov (ID NCT04218968). The 2.5 cut-off was used in sensitivity analyses, in line with population-based studies that report optimal cut-offs between 2.0 and 2.5 [26]. Based on these cut-offs, subgroups of patients with and without insulin resistance (IR+, IR−) were identified. Adherence to a healthy lifestyle and dietary pattern was evaluated using validated questionnaires administered at baseline. Adherence to the Mediterranean diet was assessed through the PREDIMED-based 14-item Mediterranean Diet Adherence Screener [27], which captures the frequency of key food groups (olive oil, fruits, vegetables, fish, legumes, nuts, and moderate wine consumption) and yields a total score ranging from 0 to 14, with higher scores indicating greater adherence.

**Statistical analysis:** Data on insulin resistance, as measured by HOMA-IR, in levodopa-naïve PD patients are currently limited. Consequently, formal sample size calculation was not feasible, and this study was conducted as a small exploratory case–control investigation to assess the prevalence of peripheral insulin resistance in early PD. Post hoc power analysis was subsequently performed for the difference in means of HOMA-IR between groups (primary outcome). Patients were recruited based on practical availability. Variables were expressed as counts (percentages) when categorical and as means (standard deviation, SD) when continuous. The normality of data was assessed using the Shapiro–Wilk test. Between-group comparisons (PD vs. HC) of continuous variables were performed using the independent samples *t*-test or the non-parametric equivalent Mann–Whitney test, as appropriate. The χ^2^/Fisher’s exact tests were employed for categorical data. Analysis of covariance (ANCOVA) on log-transformed dependent variables was used to adjust for relevant covariates, specifically age, sex, and BMI (partial η^2^ was reported as a measure of effect size). To evaluate the robustness of group effects and the potential influence of dopaminergic therapy, a sensitivity analysis was performed, excluding patients receiving dopaminergic treatment, using the same ANCOVA model. Correlation analyses were performed using Spearman or Pearson’s, as appropriate; partial correlations adjusted for age and BMI were also computed. All tests were two-tailed, and the significance level was *p* < 0.05. Analyses were performed using SPSS version 25 (IBM Corporation, Armonk, NY, USA) and GraphPad Prism version 8 (GraphPad Software Inc., San Diego, CA, USA).

## 3. Results

**Study population:** We included 60 participants: 20 patients with PD and 40 HC. Mean age was 63.8 ± 6.7 years in PD and 64.5 ± 6.8 years in HC (*p* = 0.72); 65% were male in both groups (*p* = 1.00). BMI was comparable between groups (*p* = 0.62). Regarding lifestyle factors, using a 14-item Mediterranean diet screener (PREDIMED-based; range 0–14), adherence was modestly higher in PD (median [IQR] 9 [8,9]) versus controls (8 [7,8,9]). High adherence (≥9 points) occurred in 50% (10/20) vs. 30% (12/40), not reaching statistical significance (*p* = 0.13). Concerning disease-relevant measures, mean disease duration in PD was 1.95 ± 1.05 years; baseline motor burden was mild (UPDRS-III 12.45 ± 7.56; 55% had a HY stage = 2, and most patients were tremor dominant (65%). Six of 20 (30%) PD patients were on antiparkinsonian therapy (none on levodopa): one patient on DAs monotherapy (pramipexole and ropinirole), two patients on MAO-BIs monotherapy (selegiline), and three on a combination of both DAs and MAO-BIs. None of the participants reported a family history of PD. Baseline clinical-demographic, lifestyle, disease-relevant characteristics, and metabolic measures in our cohort are shown in Table 1 and Table 2.

**Figure 1:**
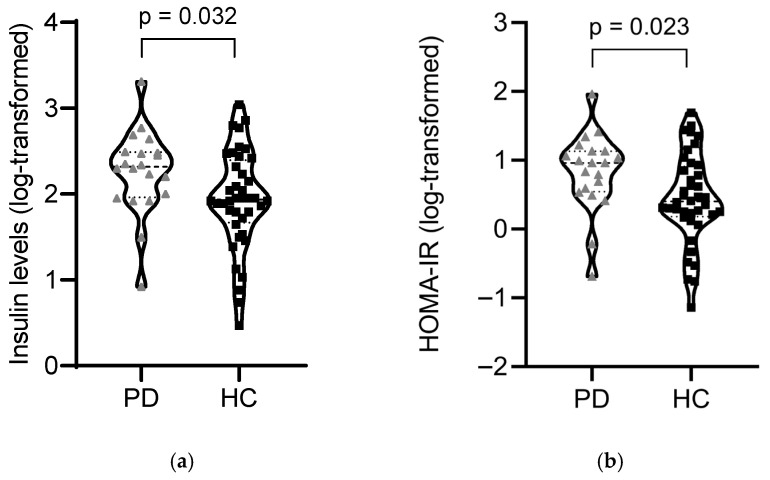
Estimated marginal means (ANCOVA analysis adjusting for age, sex, and BMI) for insulin (**a**) and HOMA-IR (log-transformed) (**b**) by PD and HC groups. Triangles and squares represent PD and HC participants, respectively. Dashed lines indicate median values, and dotted lines indicate quartiles.

**Metabolic Profile and Relationship with Clinical Measures**: With HOMA-IR ≥ 2.0, IR prevalence was 70% (14/20) in PD vs. 32.5% (13/40) in controls (OR = 4.85, 95% CI 1.52–15.50, *p* = 0.012). With HOMA-IR ≥ 2.5, IR prevalence was 55% (11/20) vs. 25% (10/40; OR = 3.67, 95% CI 1.18–11.41, *p* = 0.043). Compared with HC, PD showed higher fasting insulin (10.7 ± 5.18 vs. 8.02 ± 4.38 µIU/mL, *p* = 0.020) and higher HOMA-IR (2.63 ± 1.40 vs. 1.89 ± 1.21, *p* = 0.014); fasting glucose was higher but non-significant (*p* = 0.076). Post hoc power analysis for the difference in mean HOMA-IR between groups indicated an effect size of Cohen’s d = 0.57 and a corresponding power of 53%. The alanine aminotransferase (ALT) was lower in PD (20.9 ± 9.1 vs. 26.2 ± 11.1 U/L, *p* = 0.047). Differences in other routine biochemistry (lipids, HbA1c, thyroid/vitamin panels, renal/liver tests) were not significant. To verify whether differences in fasting insulin and HOMA-IR remained statistically significant after controlling for relevant covariates, ANCOVA models were employed. In ANCOVA on log-transformed outcomes (adjusted for age, sex, BMI), the group effect remained significant for both HOMA-IR (F value = 5.447, *p* = 0.023, partial η^2^ = 0.090) and insulin (F value = 4.866, *p* = 0.032, partial η^2^ = 0.081) (see Figure 1); as expected, BMI was also independently associated with both metabolic measures (HOMA-IR: F = 4.822, *p* = 0.032, partial η^2^ = 0.081; insulin: F = 5.089, *p* = 0.028, partial η^2^ = 0.085). A sensitivity analysis was then conducted, excluding patients receiving dopaminergic treatment (14 PD patients and 40 HC). The ANCOVA model adjusted for age, sex, and BMI confirmed a significant difference between groups for both HOMA-IR (F = 7.030, *p* = 0.011, partial η^2^ = 0.128) and insulin levels (F = 6.157, *p* = 0.017, partial η^2^ = 0.114). Notably, the group effect was not significant for glucose and ALT levels.

In PD, we found inverse correlations between disease duration and both HOMA-IR (ρ = −0.46, *p* = 0.043) and insulin (ρ = −0.50, *p* = 0.024), which were not retained after partial-correlation adjustment for age and BMI. Notably, we found a non-significant trend between HOMA-IR/insulin levels and BMI (respectively, ρ = 0.39, *p* = 0.090; ρ = 0.41, *p* = 0.074) and age (ρ = 0.38, *p* = 0.101). No significant correlations were observed between metabolic indices and disease severity scores or LEDD. In HC, metabolic measures correlated as follows: HOMA-IR with BMI (ρ = 0.43, *p* = 0.006) and age (ρ = 0.36, *p* = 0.021); insulin with BMI (ρ = 0.38, *p* = 0.015); glucose with BMI (ρ = 0.47, *p* = 0.002) and age (ρ = 0.39, *p* = 0.013); HbA1c with BMI (ρ = 0.49, *p* = 0.001) and age (ρ = 0.41, *p* = 0.009). In line with these results, when considering between-group exploratory comparisons in patients with IR (according to a HOMA-IR ≥ 2), no significant differences were observed in several clinical-demographic and lifestyle measures. However, a markedly higher proportion of IR+ patients had a BMI ≥ 25 (71.4%) compared with none in the IR− group (*p* = 0.011). A trend for elevated ferritin levels was also detected in IR+ vs. IR− patients (277.09 ± 326.41 vs. 112.30 ± 154.07 mcg/L, *p* = 0.070). When applying the alternative HOMA-IR threshold of ≥2.5, a similar trend for ferritin persisted (*p* = 0.087), whereas the association with BMI was no longer significant, supporting the use of the ≥2 cut-off for its greater sensitivity in detecting metabolically impaired subgroups, particularly in small exploratory cohorts.

## 4. Discussion

In this case–control study of 60 participants (20 levodopa-naïve PD; 40 controls), we observed the association between metabolic dysfunction and PD to clarify whether an altered metabolic status is linked to the disease and its early clinical features. Our findings demonstrate a higher metabolic burden in PD characterized by elevated fasting insulin. HOMA-IR was altered in 70% of patients with PD, while only 32.5% (13/40) of the controls had an altered HOMA-IR. Importantly, the robustness of group differences in insulin resistance indices was further supported by a sensitivity analysis excluding dopaminergic-treated patients. In this subsample, the group effect on both fasting insulin and HOMA-IR remained statistically significant after adjustment for age, sex, and BMI, with moderate effect sizes. These findings suggest that the observed metabolic alterations in the PD group are not substantially influenced by dopaminergic therapy, but rather likely reflect intrinsic pathophysiological differences. Notably, similarly high rates of previously unrecognized insulin resistance have been reported in non-diabetic PD cohorts [25], although conflicting results have been observed in de novo patients [28]. Moreover, the presence of a broader dysmetabolic profile is consistent with epidemiological evidence linking type 2 diabetes and metabolic syndrome to an increased risk of incident PD and to worse clinical trajectories among affected individuals [2,15,25].

Our results are consistent with a substantial body of epidemiological evidence linking insulin resistance, T2DM, and metabolic syndrome to an increased risk of PD. Recent meta-analyses have shown that individuals with diabetes or prediabetes have a significantly elevated risk of developing PD, with pooled estimates indicating a 20–40% higher risk compared to non-diabetic counterparts [2]. Likewise, a large meta-analysis on metabolic syndrome reported a comparable increase in PD risk, further supporting the role of systemic metabolic dysfunction as a predisposing factor [15]. Beyond glucose metabolism, obesity and dyslipidemia have also been variably implicated, with midlife obesity and low HDL cholesterol repeatedly identified as potential contributors to PD incidence [4]. In addition to epidemiological data, recent mechanistic reviews have provided insight into the biological plausibility of these associations. Ruiz-Pozo et al. highlighted how insulin resistance can directly contribute to dopaminergic vulnerability through multiple pathways, including impaired insulin/PI3K/Akt signalling, mitochondrial dysfunction, increased oxidative stress, neuroinflammation, and altered protein clearance [5]. The overlap between metabolic dysfunction and PD is supported by converging pathogenic pathways. Insulin resistance emerges as a central link: impaired insulin/PI3K/Akt signalling disrupts neuronal glucose utilization, promotes α-synuclein aggregation, increases mitochondrial stress, and alters synaptic plasticity [5,29]. In parallel, mitochondrial dysfunction leads to reduced ATP production and increased ROS, heightening dopaminergic vulnerability. Additionally, defective mitochondrial quality control/mitophagy (e.g., PINK1/Parkin pathways) may further exacerbate bioenergetic failure [6,7,30]. Impairment of autophagy-lysosome function and proteostasis, as well as endoplasmic reticulum stress, may also converge to promote dopaminergic cell death [31,32]. Furthermore, several downstream proteins of insulin signalling may be involved in the pathophysiology of PD. For instance, forkhead box O (FOXO) transcription factors are linked to metabolic alterations, tyrosine hydroxylase (TH) levels [33], and α-synuclein accumulation [34]. In this regard, in glucosylceramidase beta 1 (GBA1)-associated models, dysregulated insulin pathways and FOXO1 overactivation promoted dopaminergic neuron loss, while pioglitazone showed neuroprotective effects [35]. These findings indicate that central insulin resistance may directly contribute to PD pathogenesis, independent of peripheral metabolic alterations. However, peripheral and central insulin resistance likely represent interconnected phenomena, as systemic metabolic dysfunction can impair brain insulin signalling and exacerbate neurodegenerative processes. Our findings of increased peripheral IR in early PD therefore support a broader metabolic susceptibility underlying the disease.

Beyond intracellular mechanisms, systemic low-grade inflammation (typical of obesity and metabolic syndrome) can amplify neuroinflammatory cascades via cytokine signalling and blood–brain barrier (BBB) dysfunction [9]. In our population, the higher prevalence of hyperferritinemia in IR+ subjects may reflect the contribution of increased oxidative stress and low-grade inflammation, conditions frequently associated with hyperferritinemia [36], to the IR observed in the PD group. Altered iron metabolism represents not only a marker of systemic inflammatory status but also an active contributor to cellular damage through iron-dependent mechanisms that disrupt adipokine and hepcidin secretion. This dysregulation promotes iron deposition in crucial regions of the central nervous system, such as the substantia nigra, leading to impaired autophagy. In addition, ferritin levels are linearly associated with disorders of glucose metabolism, including diabetes. Accordingly, our findings are consistent with accumulating evidence linking increased metabolic burden to altered iron metabolism and iron-mediated inflammation, processes that may converge in PD pathophysiology [37,38,39,40].

Finally, alterations of the gut microbiota are linked to intestinal inflammation, increased permeability, and neuroactive metabolites that may facilitate α-synuclein pathology along the gut–brain axis [41]. Previous studies have suggested a link between the liver’s ability to metabolize potentially toxic compounds and the risk of PD. Notably, impaired hepatic metabolism of xenobiotics, modulated by CYP2D6 genetic variants, is more prevalent among patients, particularly those with juvenile-onset [42]. Other evidence showed significantly reduced metabolism and elimination of the hydrocarbon solvent n-hexane and its metabolites compared with age- and sex-matched healthy controls, suggesting that impaired n-hexane catabolism—worsened by ageing and smoking—might contribute to PD pathogenesis [43].

Evidence suggests an association between IR and poorer cognitive performance in PD, as assessed by MoCA and MMSE scores [44]. Moreover, cognitive decline has been linked to autonomic dysfunction, with hypoglycemia (particularly nocturnal episodes) potentially contributing to this association [45]. Nonetheless, no significant association was observed in our cohort between insulin levels and disease severity, as quantified by motor scores and non-motor symptom assessment; however, the absence of a formal cognitive assessment may have limited the interpretation of these findings.

### Strengths and Limitations

This study has several strengths, including (i) the comprehensive and standardized assessment of clinical and metabolic factors; (ii) the selection of a cohort that was entirely levodopa-naïve, with most patients also naïve to other dopaminergic therapies, allowing for evaluation of metabolic status without relevant confounding treatment effects; (iii) the case–control design with a careful selection of age-, sex- and BMI-matched controls.

Nevertheless, some limitations warrant consideration. First, the observational, case–control design precludes any causal inference. Second, metabolic status was assessed only at baseline without repeated measurements, limiting our ability to capture within-person changes or dynamic trajectories of dysmetabolism. Additionally, none of the patients had a medical history or clinical features suggestive of other metabolic or endocrine disorders associated with IR. However, rarer causes, such as the presence of anti-insulin receptor autoantibodies, were not actively excluded during participant selection. Moreover, familial or genetic predisposition to IR, which may act independently of lifestyle factors, was not assessed. Similarly, monogenic forms of PD were not formally excluded; however, the recruitment age threshold (>50 years) and the absence of reported family history of parkinsonism, reduced the likelihood of including monogenic cases. Third, although only 6/20 patients were on DAs/MAO-BIs, and therapy was suspended for ≥12 h before metabolic assessment to prevent acute pharmacological interference, the potential for chronic effects of dopaminergic therapy on systemic metabolism cannot be fully excluded. Nonetheless, the sensitivity analysis excluding dopaminergic-treated patients yielded comparable results, suggesting that the observed differences were not significantly influenced by medication use. Additionally, the modest sample size limited statistical power (post hoc analysis for the difference in mean HOMA-IR between groups indicated a power of 53%). This was mainly due to patient recruitment at the Parkinson Institute of Milan (ASST Gaetano Pini-CTO), a tertiary referral centre for PD, where most patients are referred for evaluation in the context of device-aided therapies. Consequently, only a limited number of early-stage patients were available for study inclusion. Moreover, while diagnoses were made by experienced movement disorders specialists following established criteria, some degree of diagnostic uncertainty remains, as atypical parkinsonian syndromes may clinically overlap with PD in the early phases. Finally, recruiting controls from routine health assessments may introduce a healthy-volunteer bias [46], potentially underrepresenting comorbidities and yielding a more favourable metabolic profile than in the source population, which could attenuate observed between-group differences.

## 5. Conclusions

In conclusion, our work supports the hypothesis that the association between PD and insulin resistance is not merely driven by secondary factors (age, BMI) but is likely part of a wider disorder at the intersection between neurodegeneration and systemic metabolic dysfunction. From a translational perspective, these insights strengthen the rationale for repurposing metabolic drugs and implementing lifestyle interventions as potential adjunctive strategies to dopaminergic therapy, supported by emerging clinical data on GLP-1 receptor agonists [10,11] and exercise/nutritional interventions [13,14]. Future studies should confirm these associations in larger, longitudinal cohorts and evaluate whether improving metabolic health can effectively slow PD progression. In summary, addressing metabolic dysfunction in PD may open new opportunities for risk stratification, personalized therapy, and disease modification, ultimately improving patient outcomes in this complex multisystem disorder.

## Figures and Tables

**Table 1:** Characteristics and metabolic measures in Parkinson’s disease (PD) patients and healthy controls (HC).

Variable	PD (n = 20)	HC (n = 40)	*p*-Value
Clinical-demographic characteristics			
Age, years	63.8 ± 6.7	64.5 ± 6.8	0.720
Male sex	13 (65%)	26 (65%)	1.00
BMI, kg/m^2^	25.50 ± 3.64	25.06 ± 3.40	0.620
Lifestyle factors			
Active smoking	1 (5%)	4 (10%)	0.147
Alcohol consumption	6 (30%)	15 (37.5%)	0.099
Alcohol intake, gr/week	151.2 ± 134.8	55.6 ± 38.3	0.863
Physical activity	16 (80%)	19 (47.5%)	0.664
High Mediterranean diet adherence (≥ 9)	10 (50%)	12 (30%)	0.130
Comorbidities			
Arterial hypertension	8 (40%)	12 (30%)	0.237
Dyslipidemia	5 (25%)	6 (15%)	0.096
Obesity	1 (5%)	1 (2.5%)	0.078
Therapy			
Antiplatelet	3 (15%)	2 (5%)	0.148
Anti-hypertensive	8 (40%)	11 (27.5%)	0.201
Statin	4 (20%)	6 (15%)	0.332
Biochemical and Metabolic Profile			
Fasting glucose, mg/dL	97.50 ± 10.92	92.30 ± 12.74	0.076
Fasting insulin, µIU/mL	10.70 ± 5.18	8.02 ± 4.38	**0.020**
HOMA-IR	2.63 ± 1.40	1.89 ± 1.21	**0.014**
HOMA-IR ≥ 2.0	14 (70%)	13 (32.5%)	**0.012**
HOMA-IR ≥ 2.5	11 (55%)	10 (25%)	**0.043**
Hemoglobin, g/dL	14.5 ± 1.20	14.34 ± 1.30	0.832
Platelets, 10^9^/L	230.7 ± 49.4	236.1 ± 72.21	0.884
ALT, U/L	20.9 ± 9.1	26.2 ± 11.1	**0.047**
AST, U/L	23.28 ± 6.08	25.68 ± 7.30	0.263
GGT, U/L	21.65 ± 13.78	17.68 ± 8.27	0.251
Ferritin, ng/mL	227.66 ± 291.8	227.08 ± 151.43	0.172
HbA1c, mmol/mol	36.84 ± 4.63	37.43 ± 4.39	0.961
Creatinine, mg/dL	0.92 ± 0.14	0.94 ± 0.21	0.974
Urea, mg/dL	37.15 ± 8.46	37.51 ± 13.64	0.514
TSH, µIU/mL	2.08 ± 1.21	2.25 ± 1.19	0.485
Total cholesterol, mg/dL	177.4 ± 35.52	190.85 ± 43.15	0.299
LDL, mg/dL	99.53 ± 34.61	112.20 ± 37.16	0.727
Triglycerides, mg/dL	93.7 ± 48.18	91.33 ± 39.61	0.627
Vitamin B12, pg/mL	459.8 ± 291.26	438.20 ± 134.60	0.343
Folic acid, ng/mL	9.32 ± 6.80	10.24 ± 15.39	0.780
Vitamin D, ng/mL	29.39 ± 12.46	29.84 ± 12.13	0.830
Omocysteine, µmol/L	15.42 ± 6.70	12.58 ± 4.28	0.134
Uric acid, mg/dL	4.92 ± 1.21	4.99 ± 1.04	0.800

Values are absolute count (percentage) or mean ± standard deviation. *p*-values refer to between-group comparisons. Continuous variables were compared using an independent-samples *t*-test or a Mann–Whitney test, as appropriate. Categorical variables were compared using the chi-square test or Fisher’s exact test. Significant *p*-values are highlighted in bold. Abbreviations: BMI: body mass index; HOMA-IR: homeostasis model assessment of insulin resistance; AST: aspartate aminotransferase; ALT: alanine aminotransferase; GGT: gamma-glutamyltransferase; HbA1c: glycosylated hemoglobin; TSH: Thyroid-stimulating hormone; HDL: high-density lipoprotein; LDL: low-density lipoprotein.

**Table 2:** Clinical features of patients with Parkinson’s disease.

Variables	
Disease duration, years	1.95 ± 1.05
Tremor-dominant phenotype	13 (65%)
Hyposmia	4 (20%)
Constipation	10 (50%)
Urinary dysfunction	6 (30%)
RBD	5 (25%)
UPDRS-I	1.00 ± 1.30
UPDRS-II	4.30 ± 3.67
UPDRS-III	12.45 ± 7.56
HY 1	7 (35%)
HY 1.5	2 (10%)
HY 2	11 (55%)
DA agonist monotherapy	1 (5%)
MAO-BIs monotherapy	2 (10%)
DA + MAO-BIs combination therapy	3 (15%)
LEDD DA, mg	119.25 ± 83.4
LEDD MAO-BIs, mg	100.0 ± 0
Total LEDD, mg	162.8 ± 103.6

Values are absolute count (percentage), mean ± standard deviation. Abbreviations: RBD: rapid eye movement sleep behaviour disorder; UPDRS: Unified Parkinson’s Disease Rating Scale; HY: Hoehn and Yahr scale; DA: dopamine agonists; MAO-BIs: Monoamine Oxidase B Inhibitors; LEDD: Levodopa equivalent daily dose.

## Data Availability

The raw data supporting the conclusions of this article will be made available by the authors on request.
